# A review of natural compounds to regulate platelet aggregation: molecular mechanism and research advance

**DOI:** 10.3389/fphar.2025.1537776

**Published:** 2025-06-27

**Authors:** Yi Hou, Hong Li, Luochen Zhu, Yue Li, Yue Zeng, Tian Quan, Zhangqiang Xiang, Yue Zhang, Yuan Bian, Yuxun Wei

**Affiliations:** ^1^ Pharmacy Department, Clinical Trial Institution, The People’s Hospital of Zhongjiang, Deyang, China; ^2^ West China School of Medicine, Sichuan University, Chengdu, China; ^3^ Department of Pharmacy, Nantong Tumor Hospital (Tumor Hospital Affiliated to Nantong University), Nantong, China; ^4^ Molecular Urooncology Department of Urology Klinikum rechts der Isar Technical University of Munich Ismaningerstr, München, Germany; ^5^ Phase 1 Clinical Trial Center, Deyang People’s Hospital, Deyang, China; ^6^ Department of Oncology, Xichang People’s Hospital, Xichang, China

**Keywords:** platelet aggregation, platelet activation, natural compounds, molecular mechanism, clinical trials

## Abstract

Platelets are a class of blood cells exfoliated from bone marrow megakaryocytes and important participants in the blood. Aggregation is a prominent part of the platelets involved in the hemostasis process, regulated by multiple signaling pathways. Abnormal platelet aggregation could lead to thrombosis or hemorrhagic disorders, which is closely related to the abnormal expression of receptors inside and outside platelet cells and the mis-transmission of signaling factors. In recent years, natural compounds have been shown to regulate platelet aggregation on different levels, including platelet surface receptors, intracellular signaling factors, and release reaction from platelet secretory granules, due to their structiral characteristics. However, the anti-platelet aggregation mechanism of natural compounds is not comprehensive. Therefore, we have elaborated the main pathways that affect platelet aggregation in terms of the adenosine diphosphate (ADP), the levels of cAMP and cGMP, arachidonic acid (ARA) metabolism pathway, thrombin and collagen pathways in this paper. Particularly, we reviewed various natural compounds such as glycosides, coumarins, alkaloids, and acids that affect platelet aggregation mechanisms through these pathways. This review provides a reference for the application of natural compounds in the structural modification of platelet aggregation as well as in clinical studies.

## 1 Introduction

**FIGURE 1:**
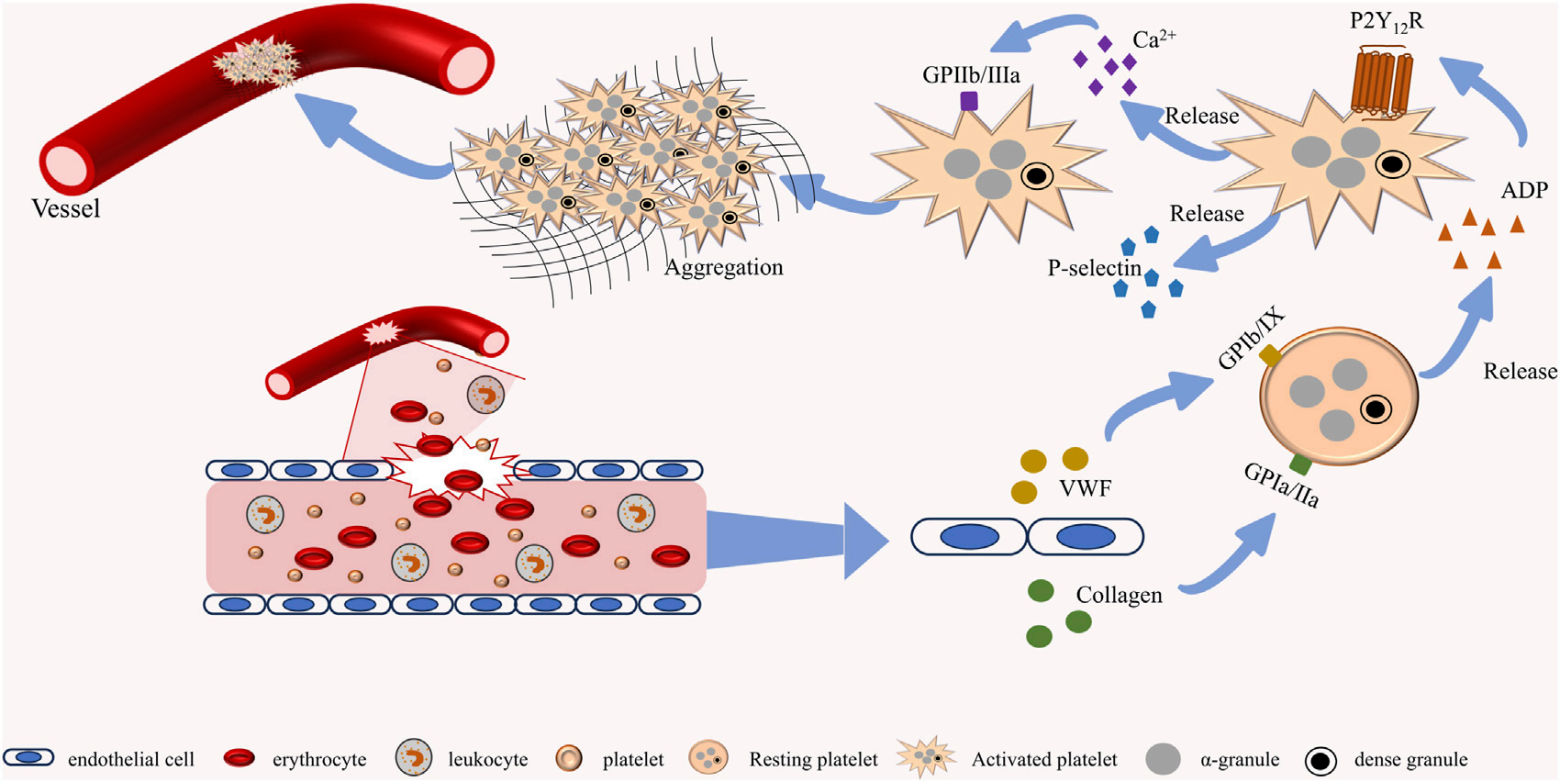
Normal platelet function. When blood vessels rupture, endothelial cells release neurotransmitters such as vWF and collagen to activate platelets. After platelet activation, dense granules and α-granules containing multiple platelet agonists are further released to promote the activation of adjacent platelets. Activated platelets undergo morphological changes and form complexes with collagen, ultimately completing the hemostasis.

Aggregation is a major physiological function of platelets. The damaged blood vessels would release signaling factors that induce platelet activation (Filkova et al., 2019; Rinder et al., 1991). Activated platelets filopodia and aggregate with one another, ultimately leading to the formation of blood clots to prevent bleeding caused by vascular injury (Dziedzic et al., 2024; Li et al., 2017). This mechanism serves as a normal defense response of the body (Figure 1). However, abnormal platelet aggregation could pose significant risks to health. Excessive aggregation of activated platelets could result in intravascular thrombosis, hindering normal blood circulation and contributing to conditions such as cerebral thrombosis, acute myocardial infarction, and atherosclerosis (Huynh et al., 2023; Joshi et al., 2022; Lordan et al., 2021). Conversely, dysfunction in platelet aggregation could impede the repair of damaged blood vessels, leading to prolonged bleeding. This dysfunction is particularly prevalent in conditions such as thrombocytopenic purpura, myelodysplastic syndrome, acute leukemia, and giant platelet syndrome (Foss and Bruserud, 2008; Markewitz and Falk, 2022; Wu et al., 2022). In response to extensive research on the mechanisms of platelet aggregation, researchers have developed various antiplatelet drugs with distinct pharmacological effects. For instance, the effects of aspirin on platelet aggregation were elucidated through studies of the arachidonic acid (ARA) metabolic pathway. Aspirin is a classic drug for preventing thrombosis in clinic (Patrono, 2024; Ridker et al., 1991). Recent advances in protein and gene detection technologies have provided a more precise elucidation of platelet function mechanisms. In conclusion, further review and analysis of these mechanisms are particularly significant for the development of new antiplatelet drugs.

Numerous natural species have significantly impacted the human health industry. *Panax ginseng* C.A.Mey. [Araliaceae; *P. ginseng radix et rhizoma*] is recognized in medicine for its rich array of active ingredients. Research has demonstrated that *P. ginseng* exhibits a variety of beneficial effects, including anti-inflammatory, antioxidant, and anti-cancer properties. Furthermore, P*anax ginseng* has been shown to enhance cognitive function, bolster immune response, and address diabetes-related conditions (Zhou et al., 2023). Additionally, *P. ginseng* possesses anti-platelet aggregation activity, which reduces the risk of thrombosis and contributes to the prevention and management of cardiovascular diseases (Hirsch et al., 2017). Notably, a variety of chemicals obtained from plants exerted antiplatelet aggregation activity, albeit at low concentrations. The pharmacologically active ingredients in herbal medicines typically include glycosides, flavonoids, alkaloids, coumarins, and organic acids (Tian et al., 2023; Vissenaekens et al., 2022; Ziegler and Facchini, 2008). In recent years, research on natural drugs has increasingly focused on the specific pharmacological mechanisms of key natural compounds. Studies of these individual natural compounds have elucidated the specific targets and signaling pathways in various diseases, providing important theoretical support for the novel drug molecules.

## 2 The mechanisms of natural compounds in antiplatelet aggregation

### 2.1 Natural compounds affect platelet aggregation by inhibiting the activity of thrombin

**FIGURE 2:**
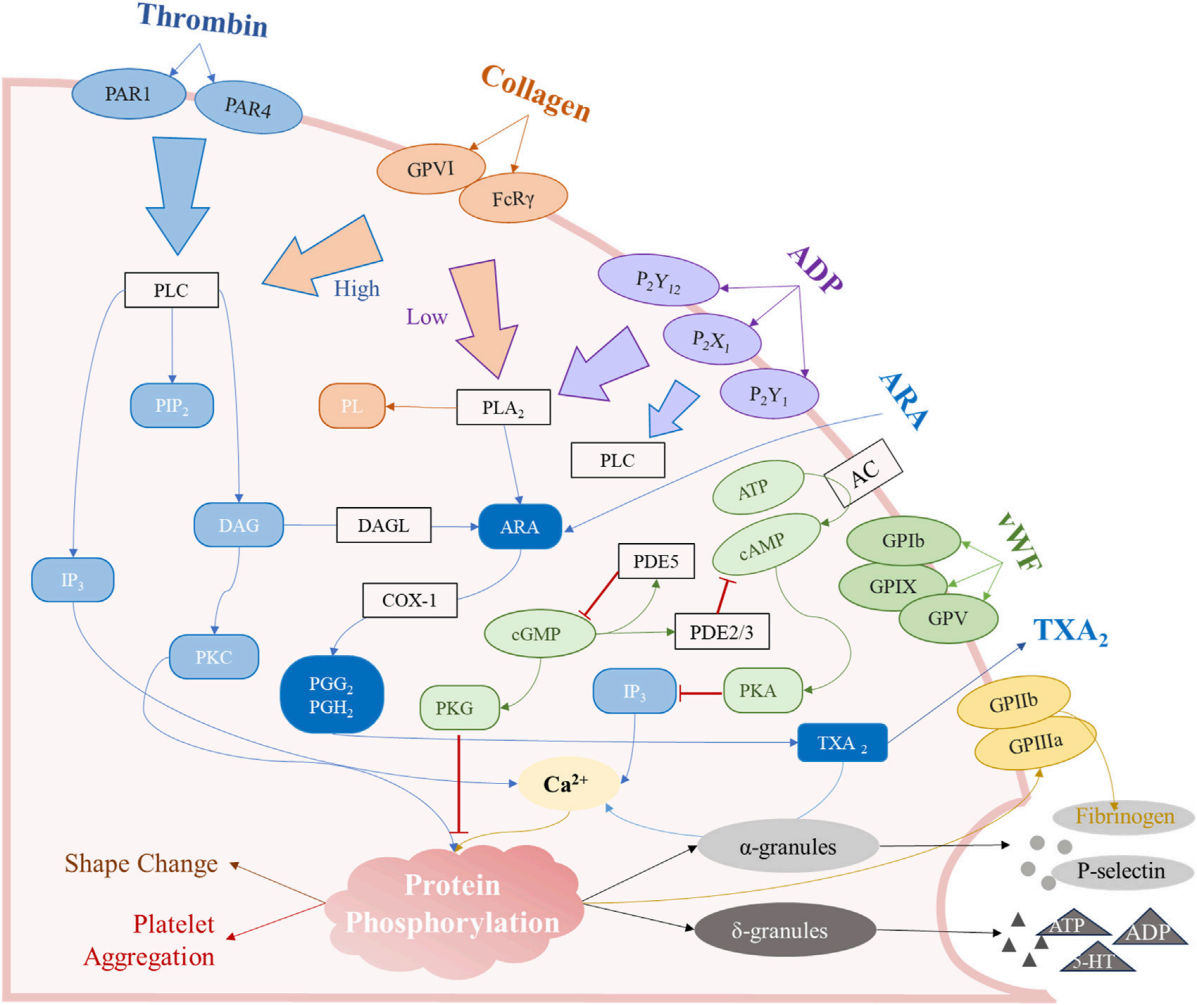
The major mechanisms of platelet aggregation. These signaling pathways are mainly involved in the arachidonic acid (ARA) metabolic pathway, the surface receptor activation pathway, the cAMP/cGMP second messenger pathway, and agonist release from intracellular granules.

Thrombin is a multifunctional serine protease produced by the cleavage of prothrombin and is recognized as a key regulator of the blood coagulation cascade, thrombosis, and platelet activation and aggregation (Davie and Kulman, 2006; Di Cera, 2008). As a pivotal enzyme that catalyzes numerous coagulation-related reactions, thrombin converts fibrinogen into fibrin, promoting the formation of insoluble cross-linked fibrin clots and thereby exerting hemostatic effects (Hulshof et al., 2021). Additionally, thrombin activates platelets, facilitating their aggregation, degranulation, and the surface expression of procoagulant lipids (such as phosphatidylserine). Thrombin binds to protease-activated receptors (PAR-1 and PAR-4) on the surface of platelets, activating Gq proteins and subsequently PLC, which promotes the generation of second messengers IP_3_ and DAG to regulate platelet aggregation (Lisman et al., 2005) (Figure 2). Consequently, natural compounds can inhibit platelet aggregation by directly inhibiting thrombin activity and by reducing the response to thrombin activation through modulation of related molecular pathways. In the prevention and treatment of thrombotic diseases, researchers have developed numerous effective agents that modulate the coagulation process by directly or indirectly inhibiting thrombin, including Argatroban, Heparin, Warfarin, Rivaroxaban, Dabigatran, and Bivalirudin (Greinacher et al., 2015). However, the clinical use of these thrombin inhibitors may lead to severe side effects, such as bleeding and allergic reactions (Van Aken et al., 2001).

Fortunately, a diverse array of substances exhibiting thrombin-inhibiting activity can be sourced from the rich repository of natural compounds. Berberine (BBR), an isoquinoline alkaloid, is a natural compound that is widely distributed across various plants, including *Coptis chinensis* Franch. [Ranunculaceae; *C. chinensis radix et rhizoma*], *Phellodendron amurense* Rupr [Rutaceae; *P. amurense bark*]. BBR possesses a diverse range of pharmacological activities, including hypoglycemic, hypolipidemic, anti-inflammatory, anti-tumor, and cardiovascular protective effects (Feng X. et al., 2019; Habtemariam, 2020; Song et al., 2020). It has been utilized in traditional Chinese medicine for thousands of years. Studies have demonstrated that BBR can inhibit thrombin-induced platelet aggregation in washed platelet samples (Wang et al., 2017). Competitive binding assays indicate that BBR binds to the same interaction sites as argatroban/thrombin. The C10 methoxy group of BBR serves as a crucial hydrogen bond acceptor, interacting with the Phe 227 and Trp 215 residues of thrombin, which aligns with the key amino acid residues in the active site region of thrombin (Mathews and Tulinsky, 1995). Furthermore, the aromatic ring A of BBR interacts with Trp-60 D of thrombin through pi-pi interactions, suggesting that Trp-60 D is a critical amino acid residue involved in the binding of BBR during the interaction process. This evidence supports the conclusion that BBR acts as a direct thrombin inhibitor.

Several natural flavonoids and polyphenols derived from herbal medicine have been recognized as thrombin inhibitors (Liu et al., 2010). The four primary biflavonoids found in Ginkgo biloba—ginkgetin, isoginkgetin, bilobetin, and amentoflavone—demonstrate significant inhibitory effects on human thrombin, with IC_50_ values ranging from 8.05 μM to 17.83 μM (Chen et al., 2019). Additionally, methanol (MeOH) and hydroalcoholic (HA) extracts obtained from the leaves of White Mangrove about *Laguncularia racemosa* (L.) C.F.Gaertn. [Combretaceae; *L. racemosa leaves*] have been shown to induce structural alterations in thrombin and diminish its activity. Among these extracts, quercetin-3-O-arabinoside (QAra) and quercetin-3-O-rhamnoside (Qn), the two glycosylated flavonoids, are identified as the most potent inhibitors of human thrombin activity (Rodrigues et al., 2015). *In vitro* experiments have demonstrated that salvianolic acid A directly inhibits thrombin. Interestingly, some natural compounds do not bind directly to thrombin but instead inhibit its activity by suppressing thrombin generation. The isocoumarin compound Sparstolonin B (SsnB) has been shown to reduce the catalytic activity of coagulation factor Xa (FXa) and the production of endothelial cells, thereby inhibiting thrombin activation (Kim et al., 2022). Additionally, various flavonoid compounds, including quercetin, quercetin-3-O-β-d-glucoside (isoquercetin), procyanidin B2, cyanidin, and silybin, also inhibit thrombin generation or directly impede thrombin activity, consequently preventing fibrin clot formation and blood coagulation (Bijak et al., 2014; Choi et al., 2016).

### 2.2 Natural compounds modulate adenosine diphosphate (ADP)-induced platelet aggregation

Adenosine diphosphate (ADP) is the most important nucleotide that induces platelet aggregation in human beings. Platelets have three main ADP receptors, including P2Y1 (Gq protein coupled receptor), P2Y12 (Gi protein-coupled receptor), and P2X1 (ligand gated ion channel) (Ding et al., 2005; Jones et al., 2014; Soulet et al., 2004). The platelet aggregation response mediated by the ADP signaling pathway is mainly regulated by P2Y1 and P2Y12, which play important roles in both normal hemostasis and pathological thrombosis. The P2Y1-mediated signaling pathway mainly affects the primary aggregation and morphological changes of platelets, such as pseudopodia (Hechler et al., 1998). The activated P2Y1 can affect the Gq protein to activate phospholipase C (PLC) with hydrolyzing inositol-4,5-diphosphate (PIP2) to generate inositol-1,4,5-triphosphate (IP_3_) and diacetylglycerol (DAG) (Vilahur et al., 2018). IP_3_ mainly affects the Ca^2+^concentration in platelets (Dolan and Diamond, 2014). DAG could further activate protein kinase C (PKC) to regulate integrin αIIbβ3, small G protein Rap1b, and further activate the Ras/Raf/MEK/ERK signaling pathway (Brose and Rosenmund, 2002). P2Y12 is a Gi protein-coupled receptor, expressed on platelet plasma membrane, that inhibits cAMP formation, ultimately leading to platelet degranulation and release of thromboxane A2 (TXA_2_), ADP, ATP, 5-HT and other active substances further promoting platelet aggregation (Damman et al., 2012; Hardy et al., 2005). The P2Y12 receptor-mediated signaling pathway mainly affects the stable aggregation and particle release of platelets. Consequently, drugs that block the downstream signaling of the P2Y12 receptor, such as clopidogrel, serve as primary anticoagulants.

**FIGURE 3:**
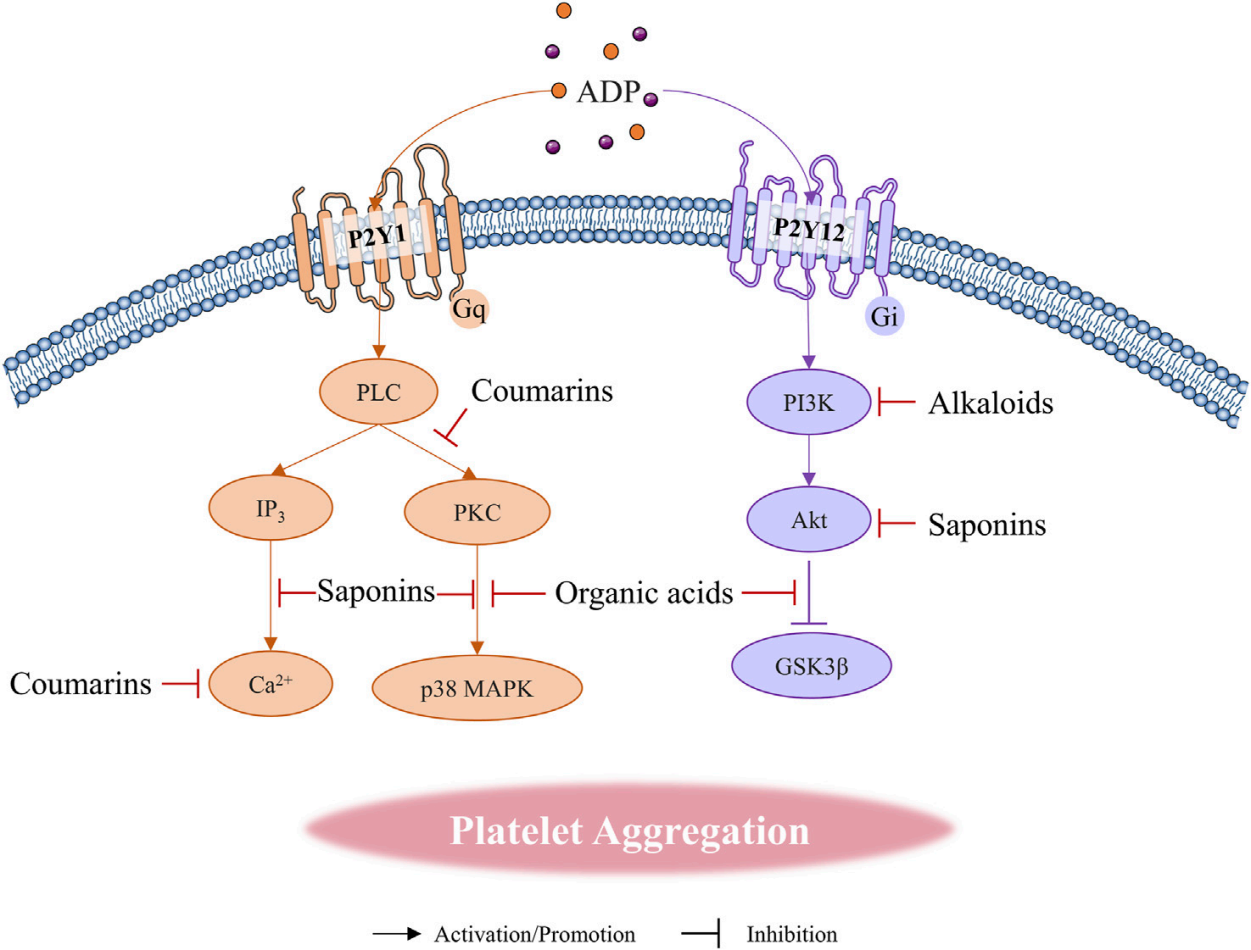
Natural compounds regulate platelet aggregation through ADP receptor pathways.

Alkaloids are a class of basic organic compounds with nitrogen-containing atoms, widely distributed in dicotyledons and gymnosperms (Bhambhani et al., 2021). Recently, Modulation of platelet aggregation through the ADP receptor pathway has been reported in alkaloids (Figure 3) such as isoquinoline, pyridines, organic, indole, and purines. Morphine, a classical isoquinoline polycyclic alkaloid primarily derived from *Papaver somniferum* L. [Papaveraceae; *P. somniferum pericarp et fruit*], was found to interfere with the antiplatelet effects of P2Y12 inhibitors (Zhang Y. et al., 2021). The PI3K β/Rasa3/Rap1 pathway is a key mechanism by which ADP activates αIIbβ3 through P2Y12 to trigger platelet aggregation. Rasa3 originates from the Ras-GAP1 family and is a major factor in maintaining the balance of GTP-Rap1 and GDP-Rap1 levels. Activated P2Y12 induces Rasa3 transfer from the cytoplasm to the cell membrane through PI3K, disrupting the above balance to increase GTP Rap1. Excessive GTP Rap1 could activate αIIbβ3 and ultimately promote platelet activation. The latest research has found that BBR could inhibit ADP-induced platelet activation and aggregation, and the mechanism is related to attenuating the activation of PI3K, thereby hindering the transfer of Rasa3 to the membrane to stabilize the quiescent state of integrin αIIbβ3 (Wang C. et al., 2021). Nicotine, as a pyridine alkaloid present in the tobacco plant, has significant neuroparalyzing effects. In addition to the effects on cardiovascular function, nicotine and other smoking products have a direct effect on platelets. Indeed, the effect of nicotine on platelet aggregation function remains controversial. Studies have found that nicotine attenuates platelet sensitivity to ADP to inhibit aggregation (Terres et al., 1989). However, other researchers have found that nicotine could promote platelet aggregation, which may be related to the duration of experimental intervention and the metabolites in human beings. For instance, nicotine-10-N-oxide has an inhibitory effect on collagen adhesion (Fahim et al., 2011; Ljungberg et al., 2013). Ephedrine, a typical organic amine alkaloid primarily derived from *Ephedra sinica* Stapf [Ephedraceae; *E. sinica Stem-herbaceous*], has been shown to inhibit the aggregation response of ADP by suppressing the exocytosis of the cytokine chemoreceptor 5 (Regulated upon Activation Normallt T-Expressed, RANTES) and the expression of P-selectin in platelet granules, which may also contribute to the induction of the hemorrhagic response (Watson et al., 2010). Colchicine mainly derived from *Colchicum autumnale* L. [Colchicaceae; *C. autumnale seeds et corm*], an alkaloid that interferes with microtubule proteins, was found to significantly inhibit platelet aggregation induced by ADP *in vitro*. The mechanism is that colchicine prevents the rearrangement of platelet organelles during aggregation and the generation of pseudopods by inhibiting the phosphorylation of associated membrane proteins such as myosin phosphatase (MYPT), LIM structural domain kinase 1 (LIMK1), and cofilin-1 (Cimmino et al., 2018). Notably, Pentoxifylline obtained by N-substituted derivatization of theobromine also inhibits ADP-induced platelet aggregation *in vitro* and *in vivo* (Magnusson et al., 2008). The metabolites of pentoxifylline *in vivo* such as 3,7-dimetyl-1 (5-hydroxyhexyl)xanthine (R-M1 and S-M1), 3,7-dimetyl -1 (4- carboxybutyl)xanthine (M4) also exhibited antiplatelet effects *in vitro*.

Saponins are a specialized class of glycoside compounds from nature, which the structure of saponins is characterized by a carbon skeleton derived from the 30-carbon 2,3-oxo-squalene precursor (de Costa et al., 2011). Some studies have reported that certain steroidal saponins and triterpenoid saponins have anti-thrombotic and anti-platelet aggregation effects, and these mechanisms involve the PI3K/Akt signaling pathway, PLC, and the effect of Ca^2+^ concentration. *Allium macrostemon* saponin, a natural saponin, derived from *A. macrostemon* Bunge [Amaryllidaceae; *A. macrostemon bulb*], inhibits ADP-induced platelet aggregation, which is related to suppressing the CD40/CD40L pathway mediated by TRAF2 ubiquitination, as well as the inhibition of downstream phosphorylation proteins such as PI3K/Akt, p38, JNK, and NF-κB (Ling et al., 2020). Meanwhile, furostanol saponins from *A. macrostemon* Bunge [Amaryllidaceae; *A. macrostemon bulb*] could inhibit ADP-induced platelet aggregation to attenuate the degree of cardiomyocyte injury. The mechanism may account for the inhibition of PI3K/Akt signaling pathway phosphorylation with platelet *in vitro* and *in vivo*, especially on Akt (Feng H. et al., 2019). This study employed the construction of a rat model, but did not conduct an examination of the standards for model establishment, such as histological verification. Meanwhile, the researchers mentioned the chromatography to separate natural compounds, we believe that increasing quantitative studies on saponin components in the mixture, such as HPLC, would better demonstrate the effect of furostanol saponins on platelet aggregation. Furostanol saponins isolated from *Anemarrhena asphodeloides* Bunge [Asparagaceae; *A. asphodeloides rhizoma*] did not inhibit ADP-induced platelet aggregation even at a concentration of 100 μg/mL, while four spiropyranol saponins as timosaponin AIII, timosaponin AII, timosaponin AIII isomers, and timosaponin III, have varying degrees of inhibitory effects on ADP-induced platelet aggregation, especially the strongest effect of timosaponin AIII (Yue et al., 2010). This result demonstrated that the structure of steroidal saponins is closely related to the anti-platelet aggregation activity. Meanwhile, the type of groups at C-3, C-15, and C-22 can influence the effect of steroidal saponins in inhibiting platelet aggregation. Previous studies have found that oleanolic acid (OA) amplifies ADP-induced platelet aggregation responses, which are linked with the activation of PLC to induce an increase in Ca^2+^ concentration, thereby promoting dense granule secretion (Kim et al., 2014; Lee et al., 2007). However, recent studies have demonstrated that OA inhibits human platelet aggregation by inhibiting the binding of αIIbβ3 to PAC-1 and the expression of P-selectin rather than ADP (Kontogianni et al., 2016). Some researchers have suggested that the opposite results in the OA study on platelet aggregation may be related to differences in platelet receptor expression with different species. Ginsenosides are the main active ingredients extracted from *P. ginseng* C.A.Mey. [Araliaceae; *P. ginseng radix et rhizoma*]. 20(S)-ginsenoside-Rg3 and 20(R)- ginsenoside-Rg3 showed different inhibitory activities against ADP and other platelet receptor-induced platelet aggregation due to the chiral character of the configurations, indicating the influence of drug spatial configuration on the platelet receptors (Lee et al., 2009). Particularly, Ginsenoside Rp3 (G-Rp3) and Rp4 (G-Rp4) derived from ginsenoside Rg1 (G-Rg1) could also inhibit ADP-induced platelet aggregation by affecting the phosphorylation of MAPK and PI3K/Akt pathways to reduce Ca^2+^ increase and aIIbβ3 activation, suggesting that different ginsenosides may act on the same signaling pathway, with varying intensities simply due to differences in functional groups and spatial structures (Irfan et al., 2018; Son et al., 2017). The latest research has found that notoginsenoside Fc (N-Fc) in *Panax notoginseng* saponins (PNS) derived from *P. notoginseng* (Burkill) F.H.Chen [Araliaceae; *P. notoginseng radix et rhizoma*] could inhibit platelet aggregation induced by thrombin, ADP, due to its ability to inhibit PLCγ2 and reduce DAG, and IP_3_(Liu et al., 2018). Interestingly, notoginsenoside Ft1 (N-Ft1) also derived from PNC, could activate PLCγ2 and affect the same signaling pathway as N-Fc to promote platelet aggregation (Liu et al., 2019). Moreover, N-Ft1 cannot induce aggregation alone, and only enhance the effects of ADP and other receptors. These two saponins are almost opposite, reflecting the duality of *P. notoginseng* (Burkill) F.H.Chen [Araliaceae; *P. notoginseng radix et rhizoma*] in affecting platelet.

Various organic acids have been demonstrated the inhibiting effects of platelet aggregation and thrombosis. Succinic acid is a major intermediate metabolite of the tricarboxylic acid cycle in aerobic life forms. The platelet surface receptor SUCNR1 (GPR91) is a succinate-mediated G-protein coupled receptor with a locus on chromosome three that is in close proximity to P2Y1 and P2Y12 genes (Ariza et al., 2012). Previous studies have shown that succinic acid can enhance the effect of platelet agonists on aggregation responses (Macaulay et al., 2007). Further research has found that succinic acid can reverse the effects of P2Y12 and P2Y1 inhibitors on ADP-mediated platelet aggregation (Spath et al., 2012). This mechanism may be related to the amplification of aggregation by succinic acid activating the relevant G-protein coupling pathway. However, citric acid, also an intermediate in the tricarboxylic acid cycle, could inhibit ADP-mediated platelet aggregation to reduce myocardial ischemia and reperfusion injury *in vivo* (Tang et al., 2013). Meanwhile, this study also demonstrates that L-malic acid has a similar inhibitory effect on platelet aggregation as citric acid. 5′- nucleotidase is an enzyme extracted from snake venom that can inhibit platelet aggregation by promoting the hydrolysis of ADP to adenosine (Saoud et al., 2017). Vanillic acid is a specific competitive agent for 5′- nucleotidase, which can reduce or even counteract the anticoagulant effect of this enzyme (Dhananjaya et al., 2006). This discovery provides ideas for the design and synthesis of new snake antivenom drugs. Gallic acid is a polyphenolic benzoic acid derived from plants. Gallic acid has been found to dose-dependently inhibit ADP-mediated platelet aggregation, which involves suppressing the expression of P-selectin and eliminating the increase of Ca^2+^. The effect of gallic acid on the phosphorylation of PKCα/p38 MAPK and Akt/GSK3β is considered to be the main mechanism in inhibiting aggregation (Chang S. S. et al., 2012). However, the concentration of gallic acid in this study was only designed to be 100, 500, 1,000 μM. Such an expanded dosing interval seems to be further optimized, and the impact of gallic acid on related proteins has not been quantitatively studied or statistically analyzed which weakens the reliability of its results. Meanwhile, this study only considered the effects of gallic acid on platelets *in vitro*. Pharmacological studies *in vivo* and linkage to thrombus models are necessary to refine this study. Rosmarinic acid, a natural phenolic acid derived from *Salvia rosmarinus* Spenn. [Lamiaceae; *S. rosmarinus stem et leaves*], could inhibit platelet aggregation induced by agonists such as ADP, correlated with inhibiting the release of Platelet-derived microvesicles (PMVs) and reducing Ca^2+^ (Chen et al., 2022). In conclusion, the Akt and p38/MAPK pathways seem to be common mechanisms with most phenolic acids in the inhibition of platelet aggregation, which suggests a direction for researchers in the new platelet drugs.

The coumarins and the derivatives have excellent anti-platelet aggregation and vasodilation effects and have been earlier applied in antithrombotic and anticoagulant therapy. Feroniellin B, isolated from *Feroniella lucida* (Scheff.) Swingle [Rutaceae; *F. lucida root*], significantly reduced platelet aggregation by 59.1% at 150 μg/mL, and the inhibitory effect is 39 times higher than that of the standard drug ibuprofen (Phuwapraisirisan et al., 2007). Otherwise, feroniellins A and C with a similar branched structure to feroniellins B have demonstrated different levels of aggregation inhibition. This oxygen-containing cyclic structure seems to be involved in the antagonism of ADP receptors, and the oxygenated pyran moiety of feroniellin B is the most effective. Sparstolonin B (SsnB) is an isocoumarin compound extracted from medicinal plants such as *Sparganium stoloniferum* (Buch.-Ham. ex Graebn.) Buch.-Ham. ex Juz. [Typhaceae; *S. stoloniferum rhizoma*] and *Bolboschoenus yagara* (Ohwi) Y.C.Yang and M.Zhan [Cyperaceae; *B. yagara Stem-tuber*]. SsnB can inhibit platelet aggregation induced by ADP, which may be related to the inhibition of PLCγ2/PKC phosphorylation and intracellular calcium increase (Kim et al., 2022). This research have effectively demonstrated the anti-aggregation effect of SsnB on human platelets and mouse models both *in vivo* and *in vitro*. In the mice model, the injection dose was only calculated and predicted based on the circulating blood volume and body weight. To explore the dose-response relationship of SsnB *in vivo*, increased pharmacokinetic studies of SsnB *in vivo* would be better. Meanwhile, Hyuganin C, a coumarin compound extracted from *Angelica sinensis* (Oliv.) Diels [Apiaceae; *A. sinensis radix et rhizoma*] also significantly inhibited the platelet aggregation induced by the abnormal increase in ADP (Mira et al., 2017; Yang et al., 2014). Six coumarin derivatives synthesized by coumarin and the isomers could all inhibit ADP-mediated platelet aggregation, among which 7-hydroxy-3-phenyl 4H-chromen-4-one has the most significant effect. The mechanism of these new coumarin derivatives involves inhibiting the activation of GPIIb/IIIa on platelets, suppressing the increase of Ca^2+^ downstream of P2Y1, and eliminating the negative regulation of P2Y12 (Lu et al., 2022).

### 2.3 Natural compounds affect aggregation by regulating the levels of cAMP and cGMP

Cyclic nucleotides serve as major second messengers in humans and play a crucial regulatory role in various cellular processes. The activation of the cyclic adenosine monophosphate (cAMP)/cyclic guanosine monophosphate (cGMP) pathway has an inhibitory effect on platelet activation and aggregation (Figure 2), with functional abnormalities strongly associated with thrombotic and hemorrhagic diseases. The balance of synthesis and catabolism in the two cyclic nucleotides maintains the normal expression of platelet function. PGI2 binds to prostaglandin receptors (IP, a G protein-coupled receptor), stimulating adenylate cyclase (AC) to convert ATP into cAMP (Reitmair et al., 2012). Conversely, activated Gi proteins inhibit AC to reduce cAMP promoting platelet aggregation (Ferreira et al., 2004). Additionally, the NO-soluble guanylate cyclase (sGC) - cGMP signaling pathway represents another critical second messenger pathway that influences platelet function (Triposkiadis et al., 2022). The sGC protein exists in the cytoplasm of platelets and could be activated by NO entering the platelets to catalyze the generation of cGMP to inhibit platelet activation (Friebe and Koesling, 2003; Zhou et al., 2025). The drugs that generate NO, such as sodium nitroprusside and nitroglycerin, could influence platelet function through this pathway (Anfossi et al., 2001; Aoki et al., 1997). The cAMP and cGMP inhibit platelet function by affecting downstream protein phosphorylation *via* protein kinases PKA and PKG, respectively. For instance, cAMP and cGMP could eliminate GPIbα-mediated platelet aggregation, which is attributed to the inhibition of the Akt pathway (Makhoul et al., 2019). Vasodilator-stimulated phosphoprotein (VASP), upon phosphorylation by PKA, further inhibits platelet activation. Meanwhile, PKG-mediated phosphorylation of substrates can hinder platelet aggregation by affecting Ca^2+^ release.

Regulating the concentration of cAMP in platelets is the main pathway by which alkaloids affect platelet aggregation. The mechanism of morphine regarding the promotion of aggregation is related to the activation of α-2- adrenoceptors in platelets as a consequence of elevating the intracellular Ca^2+^ concentration and the inhibition of adenylate cyclase to reduce the cAMP (Sheu et al., 2002). Moreover, the decrease in cAMP caused by morphine accelerates ATP release, leading to further platelet activation. Interestingly, yohimbine, an indole alkaloid from *Corynanthe johimbe* K.Schum. [Rubiaceae; *C. johimbe bark*], could inhibit platelet aggregation by blocking α-receptors to eliminate Ca^2+^ release and elevate platelet cAMP concentrations, which is a diametrically opposed pharmacological activity to morphine (Kubacka et al., 2018; Saeed and Rasheed, 2003). Indeed, research has found that yohimbine could indeed inhibit platelet aggregation caused by morphine. Matrine was found to inhibit platelet aggregation by increasing levels of cGMP. Particularly, matrine does not alter the expression of P-selectin, GPIbα, GPVI, or αIIbβ3 (Zhang S. et al., 2021). Modulation of the cAMP degradation process by affecting the activity of PDE is a major pathway by which alkaloids affect platelet aggregation, as reported in papaverine and aminophylline. Papaverine, derived from the same plant as morphine, inhibits PDE to increase cAMP concentration to reduce platelet aggregation (Zahavi et al., 1984). Meanwhile, papaverine has a direct anti-aggregation effect without inhibiting endothelium cell-dependent platelet aggregation (Az-Ma et al., 2000). Aminophylline, a clinical alkaloid used in asthma and chronic obstructive pulmonary diseases (COPD), could also inhibit platelet aggregation by inhibiting PDE to increase cAMP levels (Dow-Edwards et al., 1980; McDonald et al., 1974). Earlier studies have found that chronic administration of caffeine can enhance the inhibitory effects of prostaglandin E1 (PGE1) and 5′- N-ethylcarboxamide adenosine (NECA) on platelet aggregation, which is attributed to caffeine altering the number of adenosine receptors to promote an increase in cAMP levels (Zhang and Wells, 1990). Theobromine is a phosphodiesterase inhibitor, and a purine alkaloid widely found in tea, coffee, and chocolate (Jang et al., 2020). The administration of chocolate containing theobromine reduces platelet aggregation activated by ADP, which is related to increasing the sensitivity of platelets with theobromine to cAMP (Rull et al., 2015). In conclusion, the effects of purine alkaloids on platelet aggregation are mostly mediated through the cAMP pathway, which may be related to the purine structure of cAMP.

A few studies have reported the effect of saponins on cAMP in platelet aggregation, typically consisting of triterpenoid saponins. Ginsenoside Rk3 (G-Rk3) was found to increase cAMP in human platelets to induce phosphorylation of the cAMP-dependent kinase substrates VASP and inositol 1,4,5-trisphosphate receptor (IP_3_R), and significantly inhibit Ca^2+^ recruitment and cytoplasmic activation of integrin aIIbβ3 (Kwon et al., 2023). The main components of Panaxatriol saponins (PTS) are ginsenosides Rg1 (G-Rg1), ginsenosides Re (G-Re), and Notoginsenoside R1 (N-R1) with a total content of more than 67%. A study confirmed that PTS can inhibit platelet aggregation by increasing cAMP, and the inhibitory effect was stronger than that of PNS (Xu et al., 2021).

Currently, the studies on organic acids in platelet aggregation regarding the cAMP or cGMP pathway are still few. A new formula derived from *Cornus officinalis* Siebold & Zucc. [Cornaceae; *C. officinalis fruit*], including malic acid, succinic acid, and citric acid in the ratio of 3:2:2, inhibited platelet aggregation with a maximum inhibition rate of 82.82%. The mixture was found to increase the release of cGMP and NO from platelets, but had no effect on the concentration of cAMP (Zhang et al., 2014). Unfortunately, the specific mechanisms by which these three acids affect the NO/cGMP pathway are still unclear. Some research reports suggest that caffeic acid can promote the phosphorylation of VASP and inositol triphosphate (IP_3_) receptors by increasing cAMP levels to prevent the activation of GPIIb/IIIa receptors, thereby exerting antiplatelet aggregation (Anwar et al., 2013; Lu et al., 2015; Nam et al., 2020).

Most research has indicated that the cAMP pathway is a major pathway by which flavonoids affect platelet aggregation. For instance, the ability of quercetin and dihydroquercetin to inhibit ADP-induced platelet aggregation is closely related to reversing the increase in the content of Ca^2+^ by antagonizing ADP, thereby increasing cAMP levels (Kubatiev et al., 1999; Lanza et al., 1987). Meanwhile, some of the flavonoids, such as apigenin, quercetin, and populin, could inhibit the activity of PDE to decrease the degradation of cAMP (Balykina et al., 2024). These flavonoids could aslo increase cAMP to activate PKA activity, thereby further inhibiting platelet aggregation (Huang et al., 2021). These studies demonstrate that flavonoids increase cAMP levels in platelets through multiple pathways. Interestingly, naringen inhibits platelet aggregation by increasing the levels of cGMP rather than cAMP.

### 2.4 Natural compounds affect platelet aggregation by regulating arachidonic acid (ARA) metabolism pathway

The metabolic pathway of arachidonic acid (ARA) primarily relies on n-6 polyunsaturated fatty acids as metabolic precursors, which generate active fatty acid metabolites through the catalysis of various enzymes. These metabolites play critical roles in regulating inflammation, the hematological system, immune responses, and the respiratory system (Sonnweber et al., 2018). Cyclooxygenase (COX) is a bifunctional enzyme that catalyzes both cyclooxygenation and peroxidase reactions, metabolizing ARA released from membranes by phospholipase A2 (PLA) into prostaglandin PGG2, which is subsequently converted into prostaglandin PGH2. PGH2 serves as the major active intermediate in the prostaglandin pathway and can be further synthesized into TXA_2_ and prostaglandin I2 (PGI2) by specific enzymes (Harris and Zhang, 2011). TXA_2_ is a potent platelet-activating substance that promotes platelet aggregation, ultimately leading to thrombus formation. PGI2 is mainly synthesized and released by vascular endothelial cells. However, PGI2 can inhibit platelet aggregation by increasing the level of cAMP in platelets to inhibits the release of Ca^2+^, compared to TXA_2_. Aspirin, a well-established therapeutic agent, inhibits platelet aggregation by blocking COX-1, which in turn decreases the production of TXA_2_. Notably, PGI2 exerts physiological effects that are antagonistic to those of TXA_2_, as it is capable of inducing vasodilation and inhibiting platelet activation. Treprostinil, a synthetic analog of PGI2, has been employed in the treatment of patients suffering from pulmonary arterial hypertension and arterial thrombosis (Ghonem et al., 2011). Both TXA_2_ and PGI2 possess very short half-lives in the human body, undergoing rapid metabolism to form thromboxane B2 (TXB_2_) and 6-keto-prostaglandin F1α (6-keto-PGF) (Capone et al., 2007), respectively. Therefore, maintaining the balance between TXA_2_ and PGI2 is crucial for regulating platelet aggregation. Aspirin, recognized as the most effective antiplatelet drug, is widely employed in antithrombotic therapy due to its inhibitory effect on COX-1 *via* the ARA pathway (Desborough and Keeling, 2017).

**FIGURE 4:**
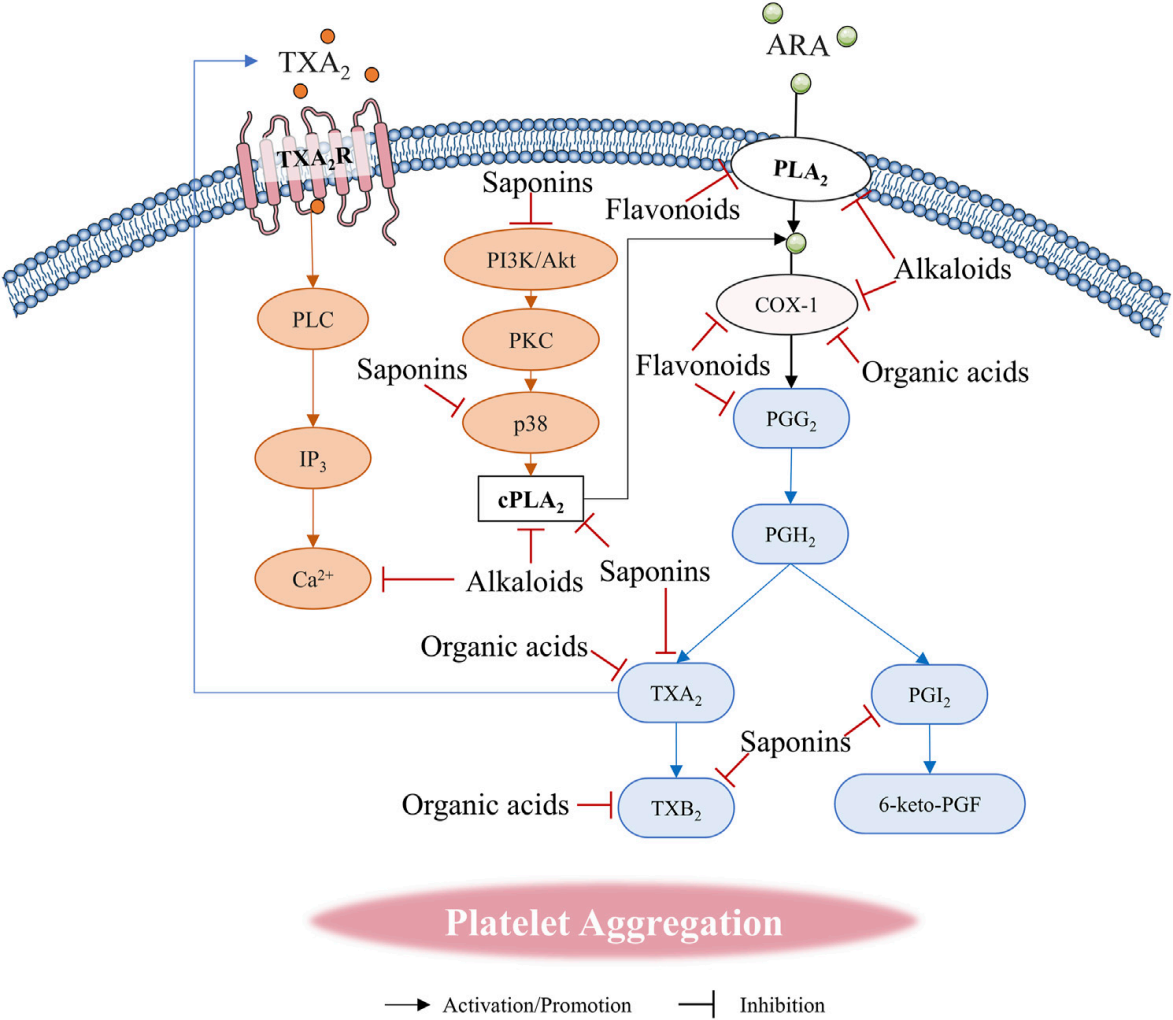
Natural compounds regulate platelet aggregation through arachidonic acid (ARA) metabolic pathway.

Recent studies have extensively reported the effects of alkaloids on platelet function, particularly regarding aggregation (Parvin et al., 2022). Certain alkaloids can influence platelet aggregation through the ARA pathway (Figure 4). Both tetrandrine (TET) and fangchinoline (FAN), which are derived from *Sinomenium acutum* (Thunb.) Rehder and E.H.Wilson [Menispermaceae; *S. acutum stem et rhizoma*]*,* inhibited PAF-mediated platelet aggregation by affecting the formation of TXA_2_, with FAN demonstrating superior efficacy compared to TET (Kim H. S. et al., 1999). Notably, these alkaloids do not interfere with the binding of PAF to its receptor. Additionally, piperine, which shares the same benzodioxole structure as BBR, also reduces the release of ARA by inhibiting cytosolic phospholipase A2 (cPLA2), thereby inhibiting platelet aggregation and suppressing TXA_2_ (Park et al., 2007; Son et al., 2014). The results suggest that the benzodioxole structure may serve as a functional group influencing platelet aggregation. Vinblastine, a bisindole alkaloid, is widely utilized in cancer treatment due to its mechanism of inhibiting tubulin polymerization. Previous studies have demonstrated that vinblastine inhibits platelet aggregation through its effects on ARA metabolism (Brammer et al., 1982). In contrast, vincristine, another bisindole alkaloid, does not affect platelet aggregation (Takano, 1981). It is important to note that tubulin is not essential for platelet aggregation (Kuntamukkula et al., 1982). Vincristine alkaloids appear to influence aggregation by disrupting the movement of granules within platelets (Sneddon, 1971). Furthermore, the study indicated that vincristine does not inhibit platelet aggregation by suppressing the microtubule pathway; instead, it affects PLA2 activity and Ca^2+^ influx, which inhibits ARA release and interferes with membrane fluidity (Hashizume et al., 1988). Specifically, vincristine exhibits a promoting effect on platelet counts at low concentrations (0–20 μg/mL) but shows an inhibitory effect on platelet aggregation at high concentrations (100 μg/mL) (Takano, 1981). Recent studies indicate that caffeine can also mitigate ARA-mediated platelet aggregation by inhibiting COX-1 and COX-2 (Hutachok et al., 2020). Furthermore, as an anticholinergic agent, anisodamine, is derived from *Anisodus tanguticus* (Maxim.) Pascher [Solanaceae; *A. tanguticus root*] has been demonstrated to be an effective inhibitor of platelet aggregation. It disrupts thromboxane synthesis by inhibiting cyclooxygenase and thromboxane synthase in the ARA pathway (Xiu et al., 1982).

Numerous recent studies have demonstrated that saponins can influence platelet aggregation *via* the ARA pathway. Ginsenosides, the primary active components extracted from *P. ginseng* C.A.Mey. [Araliaceae; *P. ginseng radix et rhizoma*], exert antiplatelet effects by interfering with associated signaling pathways and inhibiting relevant enzymes, thereby obstructing various stages of platelet aggregation (Lee et al., 2023; Zheng et al., 2019). Four dammarane-type saponins, namely, Ginsenoside Rk1 (G-Rk1), Ginsenoside Rg5, 20(S)-Ginsenoside-Rg3, and 20(R)-Ginsenoside-Rg3, have exhibited significant inhibitory activity on platelet aggregation (Zheng et al., 2019). Notably, both Rk1 and Rg5 could inhibit ARA-mediated platelet aggregation in a dose-dependent manner, surpassing the efficacy of acetylsalicylic acid (ASA). Furthermore, G-Rk1 demonstrated a pronounced inhibitory effect on collagen-stimulated human platelet aggregation, which may be attributed to its capacity to reduce TXA_2_ generation by downregulating the expression of cPLA2 (Ser505) and p38 (Shin et al., 2021). Additionally, the combination of *Panax quinquefolius* saponins (PQS) derived from *P. quinquefolius* L. [Araliaceae; *P. quinquefolius radix*] with aspirin and clopidogrel in dual antiplatelet therapy (DAPT) has been shown to enhance the antiplatelet effect in acute myocardial infarction (AMI), a mechanism attributed to the inhibition of TXB_2_. Saikosaponin A significantly inhibited ARA-induced platelet aggregation with effects comparable to those of ASA (Chang and Hsu, 1991). This data has suggested that Saikosaponin A has a therapeutic role in thrombosis. Furthermore, PQS promotes the synthesis of epoxyeicosatrienoic acid (EET) and PGI2, while simultaneously inhibiting TXB_2_, thereby further suppressing platelet aggregation (Kou et al., 2018; Wang et al., 2023). Research indicates that the inhibitory effect of PQS on platelet aggregation may involve the PI3K/Akt signaling pathway (Wang et al., 2016). Similarly, the combination of PNS with DAPT could enhance the antithrombotic effect, potentially related to the augmentation of anti-platelet aggregation, activation of the fibrinolytic system, and upregulation of 6-keto-prostaglandin F1α (6-keto-PGF1α) production (Huang et al., 2022). Additionally, a study has confirmed that PNS can inhibit platelet aggregation by decreasing the TXA_2_/PGI2 ratio (Xu et al., 2021).

Various organic acids have been demonstrated to inhibit platelet aggregation and thrombus formation (Tzakos et al., 2012), which has significant implications for the prevention and treatment of cardiovascular diseases. Acetic acid, the primary product of plant fermentation, significantly inhibits platelet aggregation mediated by ADP, collagen, thrombin, and ARA, potentially due to its inhibition of COX-1 and TXA_2_ formation (Jing et al., 2015). Phloroglucinol is widely used clinically to alleviate spasmodic pain (Corvino et al., 2023). Notably, phloroglucinol has also been demonstrated to reduce TXB_2_ formation by inhibiting the activity of COX enzymes, thereby inhibiting ARA-mediated platelet aggregation (Chang M. C. et al., 2012).

The mechanisms by which various flavonoids and their biometabolic compounds exert effects against platelet aggregation have been extensively studied. Typically, a single flavonoid compound may influence platelet aggregation directly or indirectly through multiple pathways. The ARA/COX-1/2 pathway serves as the primary mechanism by which most flavonoids inhibit platelet aggregation. Research has demonstrated that flavonoids can inhibit the activity of PLA2, thereby reducing ARA levels (Kusar et al., 2024; Ximenes et al., 2012a; Ximenes et al., 2012b). Key factors contributing to the inhibition of ARA metabolism-induced platelet aggregation by flavonoids, particularly through their effects on COX-1/2 activity, have been reported for quercetin, rutin, apigenin, and silymarin (Bijak and Saluk-Bijak, 2017; Zaragoza et al., 2022). Additionally, molecular docking studies indicate that quercetin and apigenin can interact with the Tyr385 residue on COX-1, thereby preventing COX-1 from catalyzing the conversion of ARA to the prostaglandin intermediate PGG2. This interaction may represent a crucial molecular mechanism through which flavonoids exert their effects on COX enzymes (Lescano et al., 2018; Tsiailanis et al., 2023). Interestingly, certain flavonoid compounds, such as genistein, have been found to directly inhibit platelet aggregation induced by stable TXA_2_ analogs [U46619 and 9,11-epithio-11,12-methano-thromboxane A2 (STA2)](Nakashima et al., 1991).

### 2.5 Natural compounds inhibit collagen-induced platelet aggregation

Collagen receptors in platelets include the GPIa/IIa and GPVI glycoprotein receptors. The GPIa/IIa glycoprotein receptors, also known as integrin α2β1 and CD49b, are heterodimeric proteins composed of GPIa and IIa subunits that recognize GFOGER sequences of collagen. These receptors are influenced by Mg^2+^ and Mn^2+^, which mediate adhesion and aggregation (Madamanchi et al., 2014; Pugh et al., 2010). The I domain of the α subunit in GPIa/IIa serves as a collagen-binding site and exhibits some homology with the A1 structural domain of vWF, which is essential for collagen binding in conjunction with vWF and GPIa/IIa (Depraetere et al., 1997). GPVI receptors, another major class of collagen glycoprotein receptors, play a crucial role in the early phase of platelet-collagen interactions. GPVI can activate Syk family tyrosine kinases in platelets, promoting the activation of downstream signaling proteins such as PLCγ2 and PI3K, ultimately leading to an increase in intracellular calcium levels in platelets. Initial research suggested that GPVI enhances GPIa/IIa activation, further amplifying the effects on collagen adhesion and aggregation (Zhang et al., 2023). Notably, specific antibodies against GPIa/IIa and GPVI, as well as Src family tyrosine kinase inhibitors, were utilized to confirm that GPVI and GPIa/IIa receptors activate distinct pathways, indicating that both receptors play equally important roles in platelet binding to collagen. Furthermore, the expression of these two glycoprotein receptors is closely associated with Src family kinases, protein phosphatase 1 (PP1), and Syk activation, highlighting a cooperative relationship between GPIa/IIa and GPVI (Auger et al., 2005; Sarratt et al., 2005).

Some alkaloids have been shown to inhibit platelet aggregation through the collagen pathway, mainly concentrated in indole, purine, and tropane alkaloids. Reserpine, a monoterpenoid indole alkaloid, could inhibit aggregation by inhibiting platelet adhesion to collagen and affecting granule content while accelerating platelet de-aggregation (Cazenave et al., 1977). Caffeine is widely present in coffee and tea, which is the most common purine alkaloid. The Research has suggested that caffeine still has a certain inhibitory effect on collagen-induced aggregation (Hutachok et al., 2020). Previous studies have shown that atropine, the representative of the tropane alkaloids, could inhibit collagen-induced platelet aggregation (Ishigooka et al., 1985). Unfortunately, the specific molecular mechanism of atropine in this inhibitory effect is still unclear. The tropane alkaloid cocaine from *Erythroxylum coca* Lam. [Erythroxylaceae; *E. coca leaves*] could exert inhibitory effects on collagen-stimulated platelets at high concentrations (2,500 μmol/L) *in vitro* (Cagienard et al., 2014). This mechanism may be due to the direct effect of cocaine on the binding of fibrinogen to activated platelets (Jennings et al., 1993). However, research has found that cocaine could promote platelet aggregation *in vivo*, which is the cause of some cerebral thrombosis and heart diseases (Treadwell and Robinson, 2007). Some viewpoints suggest that cocaine-mediated platelet aggregation may be related to enhanced catecholamines *in vivo* (Kloner et al., 1992).

The saponins that affect collagen-induced platelet aggregation are mainly ginsenosides. Specifically, MAPK and PI3K/Akt are common mechanisms by which these saponins inhibit the collagen pathway, and may be related to their maternal nuclear structure. Red ginseng extract enriched with ginsenoside Rg3 (Rg3-RGE) can significantly inhibit collagen-induced platelet aggregation and intracellular calcium Ca^2+^ increase in a dose-dependent manner. Meanwhile, Rg3-RGE reduces granule release induced by platelet activation and binding to fibronectin. These results also found that Rg3-RGE significantly inhibited the phosphorylation of MAPKS and PI3K/Akt pathways in aggregation (Jeong et al., 2017). G-Rk1 was found to reduce collagen-induced aggregation by inhibiting endoplasmic reticulum Ca^2+^ release and elevated αIIbβ3 activity. Meanwhile, G-Rk1 has also been shown to increase the phosphorylation of IP3RI (Ser1756), VASP (Ser157), and maintaining the unphosphorylated state of Akt at Ser473, which are typically regulated by cAMP (Shin et al., 2021). This result has indicated that G-Rk1 might inhibit platelet aggregation through the cAMP pathway. G-Rp3 regulates collagen-induced platelet activation and thrombosis by inhibiting integrin αIIbβ3 activation, MAPK signaling, Src, PLC γ 2, and PI3K/Akt activation, as well as VASP) stimulation (Irfan et al., 2018).

The influence of organic acids on platelet aggregation *via* the collagen pathway remains poorly studied. Ellagic acid inhibits the activation of Plcgamma2-DAG-PKC after collagen-mediated platelet activation, as well as reduces the activatory effect of hydroxyl radical on p38/MAPK and Akt pathways after collagen stimulation, ultimately leading to a decrease in Ca^2+^ (Chang et al., 2013). Although researchers explored the effects of ellagic acid on platelet aggregation by stimulating with collagen, thrombin, and ARA in this study, they did not include the impact of ADP, an important platelet aggregation agonist. Meanwhile, we believe that incorporating research on LY294002, a PI3K inhibitor, can better demonstrate the effect of ellagic acid on PI3K/Akt. Especially, positive control drugs in the experiment should be considered in the subsequent investigation of ellagic acid on platelet aggregation.

The GPVI receptor signaling pathway and the phosphorylation of key enzymes is the main mechanism by which some flavonoids inhibit platelet aggregation. Quercetin has been shown to inhibit collagen stimulated platelet aggregation by suppressing GPVI receptor phosphorylation (Huang et al., 2021; Hubbard et al., 2003). This research suggested that quercetin could inhibit the phosphorylation of Fc receptor γ chain (FcR γ-chain), thereby reducing the early signaling pathway response of collagen-stimulated platelet aggregation, while inhibiting the phosphorylation of Syk, LAT, and PLCγ2 to further prevent platelet aggregation response. Particularly, chrysinhas also has an inhibitory effect on collagen-mediated platelet aggregation, which is related to the inhibition of the GPVI-Syk PLCγ2-PKC-ERK2 signaling pathway after collagen stimulation.

Most of the research results show differences in the aggregation reactions of natural compounds. This may be due to differences in the stages of platelets, expression levels of platelet-related receptors, and experimental protocols. We believe that designing a standard model for natural compounds in platelet aggregation reactions is necessary, which involves sample collection time, anticoagulant pretreatment of samples, and the quantity and activity of platelets in platelet-rich plasma (PRP). In addition, quantitative standards need to be established for the concentration and activity of stimulants.

## 3 Clinic trials

### 3.1 Alkaloids

Clinical trials on alkaloids have been extensively reported (Table 1). Due to the wide application of alkaloids, clinical trials usually explore their safety. Meanwhile, due to the frequent exposure of humans to alkaloids through daily food and beverages, researchers often concentrate on examining the effects of long-term consumption of these substances on the human body. Four clinical trials have reported the effects of caffeine on platelets, which further demonstrated that caffeine inhibits platelet aggregation by enhancing cAMP levels (Choi, 2003; Lev et al., 2007; Varani et al., 2000; Whittaker et al., 2013). Moreover, the results of a 2-week trial in 45 healthy volunteers showed that caffeine potentiates cAMP effects primarily through upregulation of the adenosine A2A receptor (Varani et al., 2000). The combination of caffeine and clopidogrel has been shown to increase the inhibition of platelet aggregation in the trial involving both healthy volunteers and patients with coronary artery disease, which was also linked with elevated cAMP levels (Lev et al., 2007). The trial with 12 volunteers demonstrated that caffeine could attenuate platelet aggregation mediated by high-intensity aerobic interval training (AIT), thereby avoiding thrombus (Whittaker et al., 2013).

**TABLE 1:** Clinical research on alkaloids.

Participants	Interventions	Result	Ref.
Healthy, nonsmoking subjects	1) Caffeine 200 mg BID, 7 days 2) Caffeine 200 mg BID, 14 days 3) Caffeine 200 mg TID, 7 days	Adenosine A2A receptor is upregulated, and platelet aggregation is reduced in group 1) and 2).	Varani et al. (2000)
Healthy volunteers	Intravenous injection of 10 mg ephedrine, repeat in 15 min.	Significantly prolonged the average bleeding time by 2 min.	Flordal and Svensson (1992)
Healthy subjects	1) Morphine (5 mg i.v. bolus) + 600 mg Clopidogrel 2) Placebo (0.9% NaCl i.v. bolus) + 600 mg Clopidogrel	Morphine: Max platelet agg inhibition delayed average 2h; delayed platelet embolism inhibition under high shear; eliminated 3 times closure time prolongation by collagen ADP in widespread/rapid metabolizers.	Hobl et al. (2014)
Healthy subjects	1) Morphine (5 mg i.v. bolus) + 180 mg Ticagrelor 2) Placebo (0.9% NaCl i.v. bolus) + 180 mg Ticagrelor	Morphine does not affect ticagrelor’s effects on platelet aggregation, thrombus formation and VASP phosphorylation in whole blood.	Hobl et al. (2016)
Patients presenting with STEMI or very high-risk NSTE-ACS	1) Morphine +180 mg Ticagrelor (standard tablet or orodispersible tablet) 2) 180 mg Ticagrelor	Morphine: delayed ticagrelor onset; the percentage of HRPR is significantly higher.	Parodi et al. (2023)
Patients with acute myocardial infarction	1) Morphine (5 mg) + 180 mg Ticagrelor 2) Placebo +180 mg Ticagrelor	Morphine: reduce total exposure of ticagrelor and its metabolites, delay max plasma concentration. Increase the incidence of high platelet reactivity.	Kubica et al. (2016)
Patients with STEMI who required analgesia	1) Morphine + ASA (100 mg daily) + Ticagrelor (90 mg twice daily) 2) Fentanyl + ASA (100 mg daily) + Ticagrelor (90 mg twice daily)	Compared with Group 2), Group 1): PRU is higher; max plasma concentration of ticagrelor and its metabolite AR-C124910XX delayed and lower, total exposure reduced.	Iglesias et al. (2022)
Patients with ACS	1) Morphine + Ticagrelor (180 mg) 2) Fentanyl + Ticagrelor (180 mg)	No significant difference in fentanyl or morphine effect on ticagrelor’s platelet aggregation.	Senguttuvan et al. (2021)
Patients with STEMI or non–STEMI with persistent chest pain	1) Ticagrelor (180 mg) + PPCI 2) Ticagrelor (180 mg) + PPCI + Morphine (5 mg) 3) Ticagrelor (180 mg) + PPCI + Morphine (5 mg) + Metoclopramide (10 mg)	Compared with Group 1): Group 2) antiplatelet effect decreased; Group 3) no significant difference. Compared with Group 2): Group 3) total exposure of ticagrelor and metabolites increased.	Saad et al. (2020)
Healthy subjects	1.8 mg Colchicine	No significant effect on light transmission platelet aggregation; decrease monocyte-(MPA) and neutrophil-platelet aggregation (NPA), PAC-1 and P-selectin expression, platelet adhesion to collagen 2 h post-administration.	Shah et al. (2016)
Healthy males	1) AIT bout + Caffeine (3 mg/kg) 2) AIT bout + Placebo 3) Rest + Caffeine (3 mg/kg) 4) Rest + Placebo	AIT increases platelet function; caffeine (3 mg/kg) does not exacerbate platelet function at rest or in response to AIT.	Whittaker et al. (2013)
Queue 1: Healthy subjects Queue 2: Patients with coronary artery disease	Queue 1: 1) First week: 300 mg Clopidogrel+300 mg Caffeine (30min later), Second week: Clopidogrel + Placebo 2) First week: 300 mg Clopidogrel + Placebo, Second week: 300 mg Clopidogrel+300 mg Caffeine (after 30min) Queue 2: 3) 300 mg Caffeine+75 mg Clopidogrel (after 2.5 h)	Queue 1: Caffeine + Clopidogrel: decrease in ADP-induced platelet aggregation at 4h, activation markers at 2h, VSMC phosphorylation at 4 h. Caffeine alone: no effect on platelet markers. Queue 2: Caffeine: decrease in platelet activation markers (P-selectin, PAC-1 binding), no significant effect on platelet aggregation.	Lev et al. (2007)
Patients with ST Elevation Myocardial Infarction Treated with Pharmacological Thrombolysis (TREAT)	Background treatment: Ticagrelor or Clopidogrel 1) Morphine 2) No morphine	Morphine: higher hazard of reinfarction at 7 and 30 days, lower hazard of major bleeding.	Cantor et al. (2022)

The clinical trials of morphine mainly focus on the safety and interaction with other antiplatelet drugs, due to promoting platelet aggregation and the wide application in the clinic. A clinical trial on ST-segment elevation myocardial infarction (STEMI) suggested that morphine could increase the risk of early re-infarction and major bleeding in patients, during fibrinolysis and antiplatelet therapy for STEMI (Cantor et al., 2022). Two randomized trials reported that morphine can reduce the blood concentration of ticagrelor *in vivo*. Interestingly, the trials in healthy volunteers have shown that morphine does not affect the antiplatelet effect of tegretol (Hobl et al., 2016). A trial of acute coronary syndrome (ACS) has shown that the application of morphine did not significantly alter the in-hospital adverse events or drug side effects caused by the ticagrelor in ACS (Parodi et al., 2023). However, morphine exhibits a dual inhibitory effect on blood levels and the efficacy of tegretol in patients with acute myocardial infarction (AMI) (Kubica et al., 2016). A randomized double-blind and placebo trial has found that morphine could delay the absorption of clopidogrel and reduce the levels of the metabolites, thereby deferring and weakening the antiplatelet aggregation effect (Hobl et al., 2014). In conclusion, morphine interactions with antiplatelet drugs are related to the health status of human beings. These results remind researchers to focus on this factor with designing clinical trials.

A trial involving six healthy volunteers showed that ephedrine significantly prolonged the bleeding time of the volunteers by almost 2 min, which was associated with the competitive intervention of ephedrine on α_2_ receptors (Flordal and Svensson, 1992). Notably, smoking activates the platelets and induces an increase in procoagulant substances such as thrombin, ADP, and collagen, which exacerbates platelet aggregation. This research suggests that long-term intake of nicotine should be more concerned about altering cardiovascular function (Renaud et al., 1984). A clinical trial reported that oral administration of 1.8 mg colchicine for 2 h decreased the expression of PAC-1 and P-selectin on the platelet surface (Shah et al., 2016). However, the aggregation did not change in this trial, suggesting that the amount of impact on platelet aggregation with colchicine still needs more clinical proof.

### 3.2 Saponins

Recently years, certain clinical trials have found that saponin compounds have positive prospects in anti-platelet aggregation and anti-thrombotic effects (Table 2). Ginsenosides and Notoginsenosides have been used for the prevention and therapy of cardiovascular diseases, which are related to their regulation of platelet function and cardioprotective activity. A randomized, blinded, controlled trial for 2 months demonstrated that PNS in combination with ASA increased the inhibition of ARA metabolism-mediated expression of downstream oxidized lipids TXB_2_, PGD_2_, PGE_2_, and 11-HETE, thereby reducing platelet activation and aggregation caused by hypercoagulable states in thrombosis. Moreover, this trial also demonstrated that combined PNS could avoid ASA-induced gastrointestinal injury (Wang W. et al., 2021). A randomized controlled clinical trial for 6 months with 106 volunteers demonstrated that a commercial Chinese polyherbal preparation (CCPP) containing PNS, named Xuesaitong (Supplementary Table S1), in combination with conventional drugs, could enhance the inhibition of platelet aggregation in elderly patients with ischemic cerebrovascular disease (ICD), and the whole-blood viscosity (low-shear rate, high-shear rate) and plasma viscosity were significantly reduced, which reducing the cardiovascular events (Liu et al., 2017). For this clinical research, we believe that cross-over trials should be increased to avoid errors caused by individual differences among volunteers. Although researchers have reported adverse events related to cardiovascular diseases, we still recommend that all adverse events should be reported.

**TABLE 2:** Clinical research on saponins.

Preparation	Main components	Participants	Sample		Interventions		Time	Results (treatment group compared to control group)	Ref.
			T	C	T	C			
PNS capsule	PNS (*Panax notoginseng* saponins)	Patients with stable coronary heart disease (SCHD) and chronic gastritis	21	21	PNS (60 mg bid) + ASA (100 mg/day)	ASA (100 mg/day)	2 months	1) P-selectin expression↓, GPIIb-IIIa activation↓, platelet aggregation↓, platelet inhibition rate ↑; 2) Platelet cyclooxygenase (COX)-1 activity↓, production of TXB_2_, PGD_2_, PGE_2_, 11-HETE, the downstream oxylipids of AA/COX-1 pathway in platelets↓; 3) ASA-induced gastric mucosal injury↓, gastric level of 6,15- diketo-13,14-dihydro-prostaglandin (PG)F1α, 13,14-dihydro-15-keto-PGE_2_ and PGE_2_ from AA/PG pathway ↑.	Wang et al. (2021b)
Xuesaitong soft capsules	PNS	Patients with ischemic stroke a	1,535	1,537	Xuesaitong soft capsules (PO 120 mg bid)	Placebo (PO 120 mg bid)	3 months	The proportion of patients achieving functional independence↑.	Wu et al. (2023)
Xinyue Capsule and Fufang Chuanxiong Capsule	Total ginsenosides, ligustrazine, ferulic acid	Patients with ACS after PCI	404	404	Xinyue Capsule (PO 2 capsules tid) + Fufang Chuanxiong Capsule (PO 2 capsules tid) + Conventional treatment	Conventional treatment	6 months	The occurrence of cardiovascular events↓ (the composite of cardiac death, nonfatal recurrent MI, ischemia-driven revascularization, the composite of readmission for ACS, stroke, or congestive heart failure).	Zhang et al. (2020)
Sanchitongshu capsule	PTS (contain G-Rg1, G-Re, NG-R1)	Patients of ischemic stroke in anterior cerebral circulation	71	69	Aspirin (50 mg/day) + Sanchitongshu capsule (200 mg tid)	Aspirin (50 mg/day) + Placebo capsule	4 weeks	1) Significantly ameliorated neurological deficit and activities of daily living↑; 2) Adverse reaction occurred equally in both arms, was light to moderate.	He et al. (2011)
Xuesaitong soft capsules	PNS	Patients with ICD	50	50	Xuesaitong soft capsules (2 capsules tid) + Conventional therapy	Conventional therapy	6 months	1) Plaque size, plaque thickness, and intima-media thickness (IMT) ↓; 2) Incidence of cardiovascular events ↓.	Honyan (2017)
Xuesaitong soft capsules	PNS	Patients with acute lacunar infarction complicated by cerebral microbleeds	43	43	Xuesaitong soft capsules (2 capsules tid) + Conventional therapy	Conventional therapy	8 weeks	1) Total effective rate of treatment ↑; 2) CSS score, NIHSS score ↓; 3) Cadherin S100B expression level in serum ↓, soluble receptor for advanced glycation end products (sRAGE) level ↑.	Meihua et al. (2017)
Xuesaitong capsules	PNS	Patients with ICD	53	53	Xuesaitong soft capsules (2 capsules tid) + Conventional therapy	Conventional therapy	6 months	1) Plaque thickness, plaque size, and IMT↓; 2) Two groups of whole blood viscosity (low shear rate, high shear rate), plasma viscosity, reticulocytes, platelet aggregation rate↓; 3) Incidence of cardiovascular events↓.	Liu et al. (2017)
Compound danshen dropping pill	Salviae miltiorrhizae, Borneolum Syntheticum, Panax notoginseng	Senile angina pectoris of coronary heart disease	77	76	Compound danshen dropping pill (10 pills tid) + Conventional therapy	Isosorbide mononitrate + Conventional therapy	6 months	1) Total effective rate↑, electrocardiogram effective rate↑; 2) Incidence of adverse reaction↓.	Zhiming (2015)

Di’ao Xinxuekang (DAXXK) is a total steroid saponin extracted from the rhizomes of *Dioscorea panthaica* Prain & Burkill [Dioscoreaceae; *D. panthaica radix et rhizoma*] and *Dioscorea nipponica* Makino [Dioscoreaceae; *D. nipponica rhizoma*], with the main component being dioscin. A previous clinical trial for 6 months with 267 volunteers found that continued therapy of DAXXK significantly reduced the platelet aggregation induced by ADP and epinephrine to reduce angina events *in vivo* (Meixiu et al., 1995). The clinical trial involving 56 volunteers has demonstrated that treatment with dioscin (160mg, p.o., tid) for 8 weeks significantly reduced the rate of high platelet aggregation induced by ADP compared to the placebo group, with no significant adverse effects (Qilian and Daxin, 2006).

### 3.3 Organic acids

The source of aspirin is closely related to the natural compound salicylic acid, which was originally derived from the leaves and bark of willow trees. Research has demonstrated that salicylic acid exhibits anti-platelet aggregation activity. However, the direct use of salicylic acid presents several disadvantages, including significant gastrointestinal discomfort and other side effects that limit its clinical application (Wachtel-Galor and Benzie, 2011). Aspirin, synthesized through the acetylation modification of salicylic acid, not only retains effective anti-platelet aggregation activity but also substantially reduces clinical side effects, such as gastrointestinal irritation. Since ASA is the antiplatelet drug in various cardiovascular diseases (CVDs), its effectiveness and safety have been widely reported in clinics. Researchers have more focused on clinical studies with a dose adjustment of ASA and combination with other drugs. Thrombotic occlusion may occur in the vein graft after coronary artery bypass grafting (CABG) (Post Coronary Artery Bypass Graft Trial, 1997). A clinical trial with 110 patients found that 81 mg qid of ASA after CABG immediately inhibited TXB2 formation in serum and platelet aggregation more than 81 or 325 mg qd of ASA (Paikin et al., 2015). This result suggests that a therapeutic strategy of frequent administration of ASA may be more beneficial for the maintenance of grafts in CABG patients. ASA insensitivity in patients with CVDs is a major cause of antithrombotic treatment failure. A trial found that small doses of rivaroxaban could reverse platelet hyposensitivity to ASA in patients who have failed ASA therapy to improve efficacy. Although this trial was conducted *in vitro* with the addition of rivaroxaban, these data still provide an idea for optimizing antithrombotic therapy for ASA failure (Khan et al., 2022). Moreover, the DAPT of ASA combined with clopidogrel is the most common therapeutic schedule. A recent 6-year-long clinical trial reported that ASA combined with clopidogrel reduced the risk of neurologic deterioration in ischemic stroke (Chen et al., 2024). Phosphatidylserine (PS) is an auxiliary participant in the coagulation process on the platelet membrane. Platelet activation exposes more PS to the extra-membranous side. In this point PS exposure rather participates in platelet-depending thrombin generation, coagulation, and platelet-fibrin thrombus formation (Lentz, 2003). Ticagrelor combined with ASA reduces the extra-membrane exposure of platelet PS in patients with coronary syndromes, thus playing a positive role in prophylaxis and therapy for thrombosis (Muravlev et al., 2023).

Citric acid, as an adjuvant for most drugs, has been receiving attention in clinical practice for its effects on human beings. The trial on the effect of citric acid on hemodialysis kinetics found that citric acid can inhibit the release of serotonin to eliminate platelet activation. However, this effect does not seem to have much impact on intradialytic hypotension (IDH) (Gritters et al., 2007). Interestingly, citric acid was found to potentiate the effects of GPIIb/IIIa antagonists on platelets from healthy volunteers *in vitro* trials, especially the eptifibatide. This effect provides a rationale for citric acid in anti-aggregation and also facilitates the progress of platelet aggregation detection in the clinic (Storey et al., 1998). Likewise, the results of a trial with 15 subjects who did not respond to ASA showed that the combination of ASA and citric acid had a stronger inhibitory effect than ASA alone, depending on the improvement of platelet sensitivity to ASA by citric acid (Kaplan et al., 2000). A clinical trial involving continuous intake of caffeic acid for 1 week found that coffee exhibited different effects on different platelet agonists. For instance, coffee promotes ADP-mediated aggregation and inhibits platelet aggregation induced by collagen. Fortunately, the intake of coffee can reduce whole blood viscosity, which has positive implications for CVDs (Schumacher et al., 2011). In conclusion, more refined clinical trials are still needed to demonstrate the role of phenolic acids in coffee in platelet aggregation.

### 3.4 Flavonoids

Most of the research reported the progress of clinical trials on flavonoid-rich mixtures or extracts in antiplatelet aggregation. However, the studies in single flavonoids are still relatively scarce at present. A clinical trial found that quercetin reached 4.66 μM (±0.77) and 9.72 μM (±1.38) in human beings after 30 min of ingestion of 150 and 300 mg of quercetin-4′-O-β-D-glucoside with the oral administration, demonstrating the bioavailability of quercetin *in vivo*. Moreover, this trial demonstrated platelet aggregation was inhibited after 30 and 120 min of quercetin ingestion in human beings, which was correlated with an effect on the inhibition of the GPVI receptor pathway (Hubbard et al., 2004). Interestingly, the concentration of quercetin *in vivo* is significantly lower than the concentration at which it exerts antiplatelet aggregation effects *in vitro* (Misztal et al., 2022; Oh et al., 2012). Some studies have shown that metabolites of quercetin, such as isorhamnetin (Rodriguez et al., 2021; Stochmal et al., 2022), quercetin-3-glucuronide (Ishizawa et al., 2011; Wright et al., 2010), and 3,4-Dihydroxyphenylacetic acid (Kim et al., 1998; Kim D. H. et al., 1999), could also inhibit platelet aggregation, explaining the inconsistency between the concentrations at which quercetin exerts the pharmacological effects *in vitro* and *in vivo*. To further investigate the effects of quercetin ingestion on platelet aggregation, researchers replaced quercetin intake with onion soup rich in quercetin glycosides and found that quercetin was detectable *in vivo*. Meanwhile, platelet aggregation was inhibited as in previous studies, which was associated with inhibition of the GPVI receptor pathway (Hubbard et al., 2006). These clinical trials have demonstrated the effect of quercetin on platelet aggregation as well as *in vitro* studies. Interestingly, a trial has found that quercetin supplementation alone does not seem to affect platelet aggregation. This result may be in response to the fact that some of the quercetin prototype compounds are poorly absorbed orally in humans unable to reach effective blood concentrations to exert the pharmacological effects, which provides direction for quercetin in terms of enhancing the bioavailability (Janssen et al., 1998). Some clinical trials have investigated the role of anthocyanins in anti-platelet aggregation. In a double-blind, randomized, controlled trial with 93 participants, anthocyanins ingested as oral doses of 80, 160, and 320 mg/day, respectively, were found to inhibit platelet aggregation due to dyslipidemia. More importantly, this study found that the effect of anthocyanins on platelet aggregation was linked with the inhibition of GPIIbIIIa, ADP receptors, and the reduction of ROS levels with a pronounced dose-dependent relationship (Tian et al., 2021). Meanwhile, another double-blind, randomized, controlled trial with 16 volunteers reported a 29% reduction in ADP-induced platelet aggregation in whole blood as well as a 14% reduction in P-selectin expression after 28 days of administration of anthocyanins, which is further evidence of the antiplatelet effect of anthocyanins *in vivo* (Thompson et al., 2017). Notably, this clinical study did not report the pharmacokinetics of anthocyanins *in vivo*, which has certain limitations in demonstrating the dose-response relationship about anthocyanins. Specifically, we believe that researchers also need to report all potential adverse reactions that may occur in subjects in clinic. Interestingly, a clinical result found that epigallocatechin-3-gallate could inhibit platelet aggregation induced by ADP and collagen receptors *in vivo*, and did not affect platelet activation *per se*. Specifically, epigallocatechin-3-gallate was not associated with an increased risk of bleeding when used in conjunction with antiplatelet agents such as clopidogrel, aspirin, and tegretol (Joo et al., 2018).

### 3.5 Coumarins

Due to the widespread application of coumarin anticoagulants in cardiovascular disease, the clinical trials of coumarin drugs have focused on the effectiveness, safety, and potential for combination with other drugs (Table 3). A prospective randomized open blinded endpoint (PROBE) outcome assessment trial reported that phenprocoumon can be used as an anticoagulant in patients with atrial fibrillation and end-stage kidney disease undergoing chronic hemodialysis, which remains a need to be concerned about the risk of thromboembolic and hemorrhagic events in therapy (Reinecke et al., 2023). Dicoumarol was initially isolated from moldy sweet clover derived from *Melilotus officinalis* (L.) Lam. [Fabaceae; *M. officinalis herb*] and exhibits anticoagulant activity. However, its clinical applications are limited due to side effects, including a high risk of bleeding and a narrow therapeutic window (Sun et al., 2020). Warfarin, a derivative of dicoumarol, was synthesized by modifying its chemical structure. This modification not only preserved the anticoagulant properties but also significantly enhanced its pharmacokinetic characteristics (Duxbury and Poller, 2001). A trial reported that warfarin can significantly reduce thrombin in AMI patients both *in vitro* and *vivo*, whereas aspirin alone did not reflect this inhibitory effect on thrombin. The result explains the potential mechanism by which warfarin is superior to ASA in preventing AMI (Brodin et al., 2009). Clinical research involving 24 volunteers found that warfarin does not have a significant inhibitory effect on platelet aggregation. Moreover, elevated international normalized ratio (INR) increased collagen and adrenal-mediated platelet aggregation, even with warfarin (Chylova et al., 2021). Meanwhile, another trial demonstrated platelet hyperaggregability in three of seven subjects taking warfarin, further alerting clinical concerns about the effects of warfarin on platelets (Helgason et al., 1993). These clinical results demonstrate the complexity of warfarin on platelet aggregation in therapy, which needs to be explored even more with additional trials. Previous studies have shown that clopidogrel patients induce high on-clopidogrel platelet reactivity (HPR) to ADP, leading to a series of safety events after percutaneous coronary intervention (PCI) (Bliden et al., 2007). Clinical data demonstrated that combination therapy with phenylcoumarin significantly increased the HPR rate in patients treated with clopidogrel (Dewilde et al., 2015), which provides a clinical rationale for the considerations of combining clopidogrel with coumarin derivatives.

**TABLE 3:** Clinical research on coumarin.

Object	Sample		Interventions		Time/day	Results	Ref.
	T	C	T	C			
Patients with atrial fibrillation on chronic hemodialysis	49	48	Phenprocoumon (2.5 mg bid)	Apixaban (INR 2.0–3.0)	Median follow-up time: T (506 days); C (429 days)	No differencesin safety or efficacy outcomes.	Reinecke et al. (2023)
Patients with Nonvalvular Atrial Fibrillation Undergoing Percutaneous Coronary Intervention	980	1) 978; 2) 763	Triple therapy of warfarin, aspirin (≤100 mg once daily), and a P2Y12 platelet inhibitor (clopidogrel or ticagrelor)	Dual therapy: P_2_Y_12_ platelet antagonist (clopidogrel or ticagrelor) + 110 mg or 150 mg twice daily dabigatran	Follow-up time: ≥6 months	Dual therapy had lower rates of bleeding, irrespective of BMI. Thromboembolic event rates appeared consistent across categories of BMI.	De Caterina et al. (2020)
Survivors of acute myocardial infarction	1) 68; 2) 61	57	1) Aspirin (75 mg/d) + Warfarin (INR 2.0–2.5); 2) Warfarin (INR 2.8–4.2)	Aspirin (160 mg/d)	6 weeks	Warfarin significantly reduced the endogenous thrombin generation and the potential to generate thrombin in plasma *ex vivo*.	Brodin et al. (2009)
Patients with atrial fibrillation	100	100	Warfarin	Rivaroxaban	1 year	Patients receiving rivaroxaban treatment have a lower incidence of gastrointestinal bleeding.	Sedaghat et al. (2021)
Consecutive patients with left atrial appendage (LAA) thrombi	48	114	Phenprocoumon	Non- Vitamin K-dependent oral anticoagulants (NOACs)	1 year	1) No significant difference in the efficacy of resolution after a mean of 58 ± 42.2 (median 48) days. 2) Resolution rate of LAA-thrombi in the phenprocoumon and NOAC groups was 2/3 after 8–10 weeks. 3) Thrombi resolved more in NOAC groups after 12 weeks.	Biller et al. (2022)
Patients with recurrent venous thromboembolism	30	28	Acenocoumarol	Rivaroxaban (20 mg once a day) + Aspirin (300 mg once a day)	90 days	Recurrent thromboembolic events and minor bleeding events occurred less in the rivaroxaban plus aspirin group.	Maximiliano et al. (2023)
Patients undergoing PCI	104	1,478	Clopidogrel + Acenocoumarol	Clopidogrel	-	Concomitant treatment with acenocoumarol significantly increases platelet reactivity and the rate of HPR in patients treated with clopidogrel.	Dewilde et al. (2015)

## 4 Future perspectives and conclusion

Previous studies have demonstrated abnormal platelet aggregation could be associated with thrombosis which is a significant contributor to cardiovascular diseases such as acute myocardial infarction, stroke, atherosclerosis, and pulmonary embolism. The regulation of ADP receptors, ARA metabolic pathways, nucleotide system, collagen, and thrombin pathways regulation are the main pathways for platelet aggregation. Furthermore, the anti-platelet aggregation activity of various natural ingredients with alkaloids, organic acids, saponins, coumarins, and flavonoids has been widely reported. These ingredients could inhibit platelet-activating factors, regulate cAMP and cGMP concentrations, affect PI3K/Akt and MAPK signaling pathways, as well as interfere with adhesion molecule expression, thereby improving abnormal platelet aggregation. Despite the unique advantages of natural compounds in modulating platelet aggregation through multi-target and multi-pathway mechanisms, current research in this field still faces significant limitations. The antiplatelet mechanisms of most natural compounds remain incompletely understood, with the majority of studies focusing on isolated pathways rather than addressing the complex synergistic interactions inherent to their multi-target nature. To bridge this gap, future investigations should leverage advanced omics technologies—such as transcriptomics, proteomics, and metabolomics—to systematically elucidate the regulatory effects of natural compounds on the platelet activation network. Additionally, the metabolic profiles of many natural compounds remain poorly characterized *in vivo*, and their low bioavailability and rapid metabolic clearance further hinder clinical application. Strategies such as structural modification or formulation optimization could enhance compound stability and tissue targeting, thereby improving their druggability.

In this study, we discussed the application potential of certain natural ingredients for anti-platelet aggregation from the perspective of clinical trial effects and safety for the first time. While numerous natural compounds exhibit promising antiplatelet effects *in vitro* or in animal models, clinical evidence supporting their efficacy and safety remains limited. Many existing clinical trials suffer from methodological shortcomings, including inadequate randomization, lack of blinding, and insufficient consideration of confounding factors such as genetic variability, drug-drug interactions, and long-term safety. These limitations undermine the reliability of the findings and highlight the need for more rigorously designed clinical studies. In the clinical trials with platelets, we believe that the primary objective is to ensure that the trials are randomized and blinded. Crossover design with placebo and Reference Listed Drug (RLD) with clinical trials in healthy subjects should be set up to ensure baseline consistency of the evaluation metrics. Particularly, for the design of clinical trials in disease model groups, stratification of the age of the subjects, genetic characterization, and the introduction period of the concomitant drug should also be emphasized by the investigator. The active ingredients could exert anti-platelet aggregation effects through multiple targets and pathways, highlighting their significant potential in this area. However, future studies should aim to provide deeper insights into the various underlying mechanisms by which these compounds exhibit inhibitory activity against platelet aggregation. Additionally, the pharmacokinetic characteristics of ingredients with strong antiplatelet effects require further exploration. More high-quality clinical trials are necessary to evaluate the efficacy and safety while optimizing administration methods and dosages.
