# Supplementary material for: A review of natural compounds to regulate platelet aggregation: molecular mechanism and research advance
Source: Front Pharmacol. 2025 Jun 27;16:1537776. doi: 10.3389/fphar.2025.1537776 (PMC12245885; doi:10.3389/fphar.2025.1537776)
Supplement: Supplementary file 1 [file Table1.pdf]

Supplementary Table 1 The information about the main components contained in fomulas.

Name	Main components	Plant	Family	Ref.
Xuesaitong soft capsules	*Panax notoginseng* saponins	*Panax notoginseng* (Burkill) F.H.Chen	Araliaceae	(Wu et al. 2023, Honyan 2017, Meihua et al. 2017, Liu et al. 2017)
Di'ao Xinxuekang	A total steroid saponin	1.*Dioscorea panthaica* Prain & Burkill<br>2. *Dioscorea nipponica* Makino	1.Dioscoreaceae<br>2.Dioscoreaceae	(Meixiu et al. 1995)
Xinyue Capsule	*Panax quinquefolius* saponin	*Panax quinquefolius* L.	Araliaceae	(Zhang et al. 2020)
Fufang Chuanxiong Capsule	1.*Chuanxiong rhizome*<br>2.*Angelica sinensis radix*	*1.Conioselinum anthriscoides 'Chuanxiong'*<br>*2.Angelica sinensis* (Oliv.) Diels	1.Apiaceae<br>2.Apiaceae	(Zhang et al. 2020)
Sanchitongshu capsule	Panaxatriol saponins	*Panax notoginseng* (Burkill) F.H. Chen	Araliaceae	(He et al. 2011)
Compound danshen dropping pill	1.*Salviae miltiorrhizae radix et rhizoma*<br>2.*Panax notoginseng radix et rhizoma*<br>3.*Camphora officinarum root-crystals*	1. *Salvia miltiorrhiza* Bunge<br>2. *Panax notoginseng* (Burkill) F.H. Chen<br>3.*Camphora officinarum* Boerh. ex Fabr.	1. Lamiaceae<br>2. Araliaceae<br>3. Lauraceae	(Zhiming 2015)

He, L., X. Chen, M. Zhou, D. Zhang, J. Yang, M. Yang & D. Zhou (2011) Radix/rhizoma notoginseng extract (sanchitongtshu) for ischemic stroke: a randomized controlled study. *Phytomedicine,* 18**,** 437-42.

Honyan, J. (2017) Effect of Xuesaitong Soft Capsule on Carotid Atherosclerosis Soft Plaque and Cardiovascular Events in Elderly Patients with Cerebrovascular Disease. *Shaanxi Journal of Traditional Chinese Medicine,* 38**,** 173-175.

Liu, L., J. Chao, W. Yingying, W. Yali, L. Fangfang & W. Shuyang (2017) Effect of haemosiderin capsules on carotid atherosclerotic soft plaques and blood rheology in elderly patients with ischaemic cerebrovascular disease. *Chinese Journal of Gerontology,* 37**,** 4524-4526.

Meihua, W., W. Qiuyan, Q. C. & N. Yanfang (2017) Evaluation of the clinical efficacy of Xuesaitong soft capsules in patients with acute lacunar infarction complicated by cerebral microbleeds. *Chinese Journal of Clinical Pharmacology and Therapeutics,* 22**,** 574-579.

Meixiu, W., N. Zhonping, S. Chengxiu & L. Xiaoyan (1995) Treating 267 cases of angina pectoris of coronary heart disease with Di'ao xinxuekang. *Journal of Medical Informatics***,** 26-27.

Wu, L., H. Song, C. Zhang, A. Wang, B. Zhang, C. Xiong, X. Zhuang, Y. Zang, C. Li, Q. Fang, C. Qu, L. Wang, M. Zhang, H. Li, X. Wang, Y. Li, L. Xia, Z. Yao, Z. Nie, Y. Gao & X. Ji (2023) Efficacy and Safety of Panax notoginseng Saponins in the Treatment of Adults With Ischemic Stroke in China: A Randomized Clinical Trial. *JAMA Netw Open,* 6**,** e2317574.

Zhang, D. W., S. L. Wang, P. L. Wang, J. P. Du, Z. Y. Gao, C. L. Wang, H. Xu & D. Z. Shi (2020) The efficacy of Chinese herbal medicines on acute coronary syndrome with renal insufficiency after percutaneous coronary intervention. *J Ethnopharmacol,* 248**,** 112354.

Zhiming, H. (2015) Clinical Effect of Compound Danshen Dropping Pill in the Treatment of 77Cases With Senile Angina Pectoris of Coronary Heart Disease. *China Continuing Medical Education,* 7**,** 249-250.
